# Taiwan Rheumatology Association consensus recommendations for the management of axial spondyloarthritis

**DOI:** 10.1111/1756-185X.13752

**Published:** 2019-11-27

**Authors:** James Cheng‐Chung Wei, Chin‐Hsiu Liu, Jui‐Cheng Tseng, Lin‐Fen Hsieh, Chun‐Hsiung Chen, Hsin‐Hua Chen, Hung‐An Chen, Ying‐Chou Chen, Chung‐Tei Chou, Hsien‐Tzung Liao, Yi‐Chun Lin, Shue‐Fen Luo, Deng‐Ho Yang, Kai‐Jieh Yeo, Wen‐Chan Tsai, on behalf of the Taiwan Rheumatology Association (TRA)

**Affiliations:** ^1^ Division of Allergy, Immunology and Rheumatology, Chung Shan Medical University Hospital Institute of Medicine Chung Shan Medical University Taichung Taiwan; ^2^ Graduate Institute of Integrated Medicine China Medical University Taichung Taiwan; ^3^ Division of Allergy, Immunology and Rheumatology Taipei Tzu Chi Hospital Buddhist Tzu Chi Medical Foundation New Taipei City Taiwan; ^4^ School of Medicine Tzu Chi University Hualien Taiwan; ^5^ Division of Allergy, Immunology, and Rheumatology Kaohsiung Veterans General Hospital Kaohsiung Taiwan; ^6^ Department of Physical Medicine and Rehabilitation Shin Kong Wu Ho‐Su Memorial Hospital Taipei Taiwan; ^7^ School of Medicine Fu Jen Catholic University New Taipei City Taiwan; ^8^ Division of Allergy‐Immunology‐Rheumatology Taichung Veterans General Hospital Taichung Taiwan; ^9^ Department of Allergy, Immunology, and Rheumatology Chi Mei Medical Center Tainan Taiwan; ^10^ Department of Rheumatology, Allergy and Immunology Chang Gung Memorial Hospital‐Kaohsiung Kaohsiung Taiwan; ^11^ Division of Allergy Immunology Rheumatology Taipei Veterans General Hospital Taipei Taiwan; ^12^ Department of Allergy, Immunology, and Rheumatology Cheng Hsin General Hospital Taipei Taiwan; ^13^ Department of Rheumatology, Allergy and Immunology Chang Gung Memorial Hospital‐Linkou Taoyuan Taiwan; ^14^ Division of Rheumatology/Immunology/Allergy, Department of Internal Medicine Taichung Armed-Forces General Hospital Taichung Taiwan; ^15^ Department of Medical Laboratory Science and Biotechnology Central Taiwan University of Science and Technology Taichung Taiwan; ^16^ Division of Rheumatology/Immunology/Allergy Department of Internal Medicine Tri-Service General Hospital, National Defense Medical Center Taipei Taiwan; ^17^ Division of Rheumatology and Immunology China Medical University Hospital Taichung Taiwan; ^18^ Division of Allergy, Immunology, and Rheumatology Kaohsiung Medical University Chung‐Ho Memorial Hospital Kaohsiung Taiwan; ^19^ Department of Internal Medicine Kaohsiung Municipal Ta‐Tung Hospital Kaohsiung Taiwan

**Keywords:** ankylosing spondylitis, axial spondyloarthritis, extra‐articular manifestation, IL‐17 inhibitor, non‐radiographic axial spondyloarthritis, spinal fracture, TNF inhibitor

## Abstract

**Aim:**

To establish guidelines for the clinical management of axial spondyloarthritis that take into account local issues and clinical practice concerns for Taiwan.

**Method:**

Overarching principles and recommendations were established by consensus among a panel of rheumatology and rehabilitation experts, based on analysis of the most up‐to‐date clinical evidence and the clinical experience of panelists. All Overarching Principles and Recommendations were graded according to the standards developed by the Oxford Centre for Evidence Based Medicine, and further evaluated and modified using the Delphi method.

**Results:**

The guidelines specifically address issues such as local medical considerations, National Health Insurance reimbursement, and management of extra‐articular manifestations.

**Conclusion:**

It is hoped that this will help to optimize clinical management outcomes for axial spondyloarthritis in Taiwan.

## 1 INTRODUCTION

In recent years, several guidelines regarding the management of axial spondyloarthritis (axSpA) have been published,^1^, ^2^, ^3^, ^4^, ^5^, ^6^ predominantly by societies and experts in Europe and the USA. These guidelines represent a distillation of current knowledge on axSpA, and can provide valuable guidance to clinicians; but evidence shows that the genetic features of axSpA may vary between Asian and Caucasian patients,^7^, ^8^, ^9^, ^10^, ^11^ and when clinical issues such as limited access to biologics, limited reimbursement for treatment, limited awareness, and under‐diagnosis are taken into account, it is clear that local perspectives are needed to improve the management of axSpA. Moreover, the incidence and prevalence of tuberculosis,^12^ hepatitis B,^13^ and hepatitis C^14^ are higher in Taiwan as compared to Europe or the USA, and this may limit treatment options for Taiwanese patients, particularly regarding the use of biologics. Therefore, aspects of axSpA that have local relevance were discussed in these guidelines, and recommendations with an emphasis on improving awareness, diagnosis, management, and outcomes in Taiwanese patients were formulated. It is hoped that these guidelines will help to focus attention on under‐addressed issues in the management of axSpA, and bring a fresh perspective to the current discussion.

Axial spondyloarthritis is a chronic type of arthritis that primarily affects the sacroiliac joints and the spine.^7^ Since the publication of the 2009 Assessment of SpondyloArthritis international Society (ASAS) classification criteria,^15^ axSpA has been categorized into radiographic axSpA, which is largely synonymous with ankylosing spondylitis (AS) and presents with radiographically visible structural damage to the sacroiliac joint and axial skeleton; and non‐radiographic axSpA (nr‐axSpA), a milder form of axSpA that does not exhibit such structural damage but nevertheless imposes a heavy burden of disease.^7^, ^15^, ^16^ It has been proposed that the term “axSpA” should preferentially be used in diagnosis rather than nr‐axSpA or AS,^16^, ^17^ unless medical reasons exist to justify making a distinction.^7^, ^16^ In the spirit of this, the term “axSpA” in these guidelines encompasses both nr‐axSpA and AS.

Importantly, these guidelines seek to address less explored issues in axSpA that are important for clinical management from both a local and global perspective. There is a recommendation discussing the management of extra‐articular manifestations (EAM), primarily uveitis, psoriasis, and inflammatory bowel disease (IBD) but also encompassing other conditions that affect the lungs, kidneys, and heart of axSpA patients, with best management practices mentioned where supported by evidence. Osteoporosis and the risk of spinal fractures has been noted, since motorcycles and bicycles are one of the major modes of transport in Taiwan and can increase fracture risk, which is a serious concern as such fractures are difficult to recover from and may incapacitate a patient for life in worst‐case scenarios. Regarding treatment, recommendations for exercise have been broadened to include evidence for yoga, Tai Chi, qigong, and other types of exercise that are common in Taiwan. The latest clinical trial data for novel therapies such as interleukin‐17 inhibitors (IL‐17i) have also been included. It is hoped that the discussion of these issues will help to provide practical and relevant evidence‐based guidance to clinicians in Taiwan and beyond.

## 2 MATERIALS AND METHODS

The formulation of these guidelines was undertaken by a committee of rheumatology and rehabilitation experts on behalf of the Taiwan Rheumatology Association (TRA). The structure of the guidelines was modeled on the recently published 2016 update of the ASAS‐European League Against Rheumatism (EULAR) management recommendations for axial spondyloarthritis,^1^ and also incorporated elements from the UK National Institute for Health and Care Excellence (NICE) 2017 guideline (NG65) on the diagnosis and management of SpA in over 16s,^3^ and the British Society for Rheumatology (BSR) and British Health Professionals in Rheumatology (BHPR) guideline for the treatment of axSpA (including AS) with biologics.^4^ The objective was to establish guidelines for the clinical management of axSpA from a local perspective that would take into account issues and concerns in clinical practice that are pertinent to Taiwan. The guideline committee was convened by W.‐CT and included 15 experts in rheumatology and rehabilitation, with all members required to disclose any potential conflicts of interest prior to taking up their positions. Each committee member was assigned to conduct a systematic literature review for a specific section of these guidelines, with a special emphasis on recent studies published between 2015 and 2017 and issues not addressed by other guidelines. Collected evidence was presented to the committee for review and discussion by all members, and based on these discussion results, each member prepared the wording for the recommendations and accompanying statements in their responsible section. The recommendations and statements were then presented to the committee for discussion, voting, and revision based on the Delphi method, with a threshold of 75% required for approval of each recommendation and statement. The levels of evidence, grades of recommendation, and levels of agreement were then added to each recommendation (Table 1). Briefly, level Ia refers to evidence derived from the meta‐analysis of randomized controlled trials; level Ib refers to evidence derived from at least one randomized controlled trial; level IIa refers to evidence derived from at least one controlled study without randomization; level IIb refers to evidence derived from at least one type of quasi‐experimental study; level III refers to evidence from comparative, correlation, case‐control, or other non‐experimental descriptive studies; and level IV represents evidence from expert committee reports, opinions, or clinical experience from respected authorities. Recommendations and statements based on direct level I evidence were graded as A; those based on direct level II evidence or extrapolated from level I evidence were graded as B; those based on direct level III evidence or extrapolated from level I or level II evidence were graded as C; and those based on direct level IV evidence or extrapolated from levels I, II, or III evidence were graded as D. Levels of agreement were derived through anonymous electronic voting at a committee meeting. The final guidelines and manuscript were reviewed and approved by all committee members, and were then reviewed and ratified by the TRA Executive Committee before submission to this journal.

**Table 1:** Taiwan Rheumatology Association consensus recommendations for the management of axSpA

		LoE	GoR	LoA (%)
Overarching principles				
1	The rheumatologist serves as the main coordinator of care for axSpA, a disease with diverse manifestations that is best managed through multidisciplinary care.	—	—	100.0
2	The primary objective of axSpA treatment is to secure health‐related quality of life and normalize function for the patient to the greatest extent possible.	—	—	100.0
3	Optimal management of axSpA requires a range of treatment strategies, including non‐pharmacological treatment, pharmacological treatment, surgery, and lifestyle modification.	—	—	100.0
4	Treatment of axSpA should involve shared decision‐making between the patient and health professionals in order to achieve optimal care.	—	—	100.0
5	The management of axSpA in Taiwan is strongly influenced by the National Health Insurance reimbursement system and local health circumstances.	—	—	100.0
Recommendations				
1	Treatment for axSpA patients should be individualized according to the signs and symptoms of disease, patient characteristics, and treatment goals.	IV	D	100.0
2	The diagnosis and monitoring of axSpA disease activity should be based on clinical symptoms and signs, laboratory tests, and imaging, while the frequency of monitoring should be decided on an individual basis.	IV	D	100.0
3	axSpA patients should be treated to the clinical target (T2T) of reaching either clinical remission or at least minimal disease activity (MDA). The MDA for axSpA has not been defined yet, but achieving ASDAS < 2.1 and preferably <1.3 is recommended.	IV	D	78.6
4	Patients with axSpA should be encouraged to stop smoking and start an individualized regular exercise program as soon as possible. The program should emphasize flexibility training, especially spinal mobility exercises, but aerobic exercise, resistance training, breathing exercises, and physiotherapy are also recommended.	IIa	B	92.9
5	EAM are an important part of axSpA and should be actively evaluated and managed to improve patient outcomes.	IV	D	92.9
6	NSAIDs are the first‐line treatment to ensure symptom control for symptomatic axSpA, and it is recommended to use an optimal dose to minimize complications. Ongoing monitoring of renal function, as well as gastrointestinal and cardiovascular side effects, should be determined on an individual basis. Analgesics may be considered to treat residual pain.	Ia	A	92.9
7	Local injections of glucocorticoids to sites of inflammation and short‐term systemic glucocorticoids may be beneficial, but long‐term treatment with systemic glucocorticoids should be avoided.	IIa	B	85.7
8	Although csDMARD monotherapy is not recommended for axSpA, it can be effective against peripheral arthritis and EAM; co‐administration of csDMARDs with biologics may be beneficial in axSpA, but further evidence is needed to confirm this.	IIa	B	85.7
9	In the event of treatment failure with conventional therapy, after evaluating other causes, biologic therapy should be considered for axSpA.	Ia	A	92.9
10	Intra‐ or inter‐class switching between biologics or small molecule therapies may be considered for patients with inadequate response or who become intolerant to therapy.	Ia	A	92.9
11	In patients with refractory pain or disability and radiographically visible structural damage of the hip joint, hip arthroplasty should be considered, while corrective osteotomy may be considered for patients with disabling spinal deformity.	III	C	100.0

Abbreviations: ASDAS, Ankylosing Spondylitis Disease Activity Score; axSpA, axial spondyloarthritis; csDMARDs, conventional synthetic disease‐modifying antirheumatic drugs; EAM, extra‐articular manifestations; GoR, grade of recommendation; LoA, level of agreement; LoE, level of evidence; NSAIDs, nonsteroidal anti‐inflammatory drugs.

## 3 RESULTS

These guidelines are intended for the use of all healthcare professionals involved in the management of axSpA, including rheumatologists, physiatrists, and clinicians of other disciplines. It is also important to explain these guidelines to patients and ensure their informed participation in shared decision‐making regarding treatment and care. Considering that these guidelines are intended to focus on the management of axSpA, other aspects of the disease such as classification, diagnosis, and pathogenesis will not be discussed, unless they are relevant to treatment decisions.

As both axSpA and nr‐axSpA are relatively new concepts,^15^, ^16^, ^17^ it is inevitable that a significant proportion of the evidence in these guidelines was derived from AS patients. However, efforts have been made to identify and include evidence from studies conducted in axSpA patients, and although the term “axSpA” is used in these guidelines to refer to all patients across the spectrum of disease, the terms “nr‐axSpA” and “AS” are also used where circumstances call for greater specificity. These guidelines also address the management of EAM and other comorbidities (eg osteoporosis and fractures) in axSpA, and although the guideline committee recognizes that these issues are complex and may even deserve their own guidelines, it is important for healthcare professionals to take these into account when advising patients, selecting treatment, and evaluating risks such as drug‐drug interactions.

As with the ASAS‐EULAR guidelines,^1^ these guidelines begin with a set of overarching principles that are meant to be kept in mind throughout the management of axSpA. These principles define the main considerations, influencing factors, and best approaches regarding axSpA care in Taiwan today. The overarching principles and background statements are presented below, along with their respective levels of evidence (LoE), grade of recommendation (GoR), and levels of agreement (LoA; see Table 1).

**Overarching Principle 1: The rheumatologist serves as the main coordinator of care for axSpA, a disease with diverse manifestations that is best managed through multidisciplinary care. (LoA: 100%)**

In a recent meta‐analysis of eight studies (seven longitudinal cohort studies and one cross‐sectional study) involving 1242 nr‐axSpA patients and 2236 AS patients,^18^ it was found that the pooled prevalence of a history of EAM such as uveitis (15.9% vs 23.0%), psoriasis (10.9% vs 10.2%), and IBD (6.4% vs 4.1%) in nr‐axSpA and AS patients was comparably high, indicating that the occurrence of EAM is independent of disease severity and structural damage, and suggesting that EAM prevalence numbers previously derived exclusively from AS patients may also apply to nr‐axSpA patients. Another recent systematic review of 156 studies (involving >44 000 AS patients) that further compared the prevalence of EAM in different regions derived similar results regarding the overall pooled prevalence of acute anterior uveitis (25.8%), psoriasis (9.3%), and IBD (6.8%),^19^ although prevalence was lower in Asia (uveitis: 21.4%; psoriasis: 3.1%; IBD: 2.9%) compared to Europe and other regions. Moreover, AS patients have been reported to have higher risk of developing comorbidities as diverse as hypertension, dyslipidemia, metabolic syndrome, myocardial infarction, stroke, osteoporosis, spinal fractures, obstructive sleep apnea, gastric and duodenal ulcers, depression, apical fibrosis, non‐specific and incidental abnormalities in imaging of the lungs, spontaneous pneumothorax, nephrolithiasis, immunoglobulin A (IgA) nephropathy, and renal amyloidosis.^20^ Population‐based studies conducted in thousands of Taiwanese AS patients also found higher risk of hypertension,^21^ acute coronary syndrome,^22^ and peptic ulcers^21^ as compared to the general population. Taken together, these studies show that axSpA encompasses a diverse range of manifestations and comorbidities that require an integrated, multidisciplinary approach to effectively manage them. The selection of suitable treatment particularly requires an integrated approach in building a regimen that effectively checks musculoskeletal symptoms, EAM, and related comorbidities, all while avoiding potentially debilitating drug‐drug interactions. In such a situation, the rheumatologist, having broad knowledge of the axSpA disease spectrum and the patient's condition, represents the ideal candidate to serve as the main coordinator of care with clinicians and health professionals in other specialties. Rheumatologists should recognize this role and take a proactive approach in securing multidisciplinary support for axSpA patients to achieve better management outcomes.

**Overarching Principle 2: The primary objective of axSpA treatment is to secure health‐related quality of life and normalize function for the patient to the greatest extent possible. (LoA: 100%)**

Health‐related quality of life and physical function can be severely affected by axSpA, and it appears that the impact on quality of life and function is comparable for nr‐axSpA and AS patients, despite their differences in disease presentation and severity: a 2015 retrospective study comparing observational data from the South Swedish Arthritis Treatment Group register found no significant differences between nr‐axSpA and AS patients in visual analog scale scores for global health and pain, Bath Ankylosing Spondylitis Disease Activity Index (BASDAI), Bath Ankylosing Spondylitis Functional Index (BASFI), and EuroQol 5‐Dimensions (EQ‐5D).^23^ Impaired quality of life and physical function can be observed in the early stages of disease,^24^, ^25^ and generally worsens in later stages.^26^, ^27^, ^28^, ^29^ Moreover, daily life can be disrupted in multiple ways for axSpA patients: 53%‐76% of AS patients report having problems with fatigue^30^, ^31^, ^32^; axSpA patients are more likely than the general population to have poor sleep, decreased sleep efficiency (total sleep time divided by time in bed), lower percentage of deep sleep, increased incidence of restless leg syndrome or similar symptoms, and a higher risk of obstructive sleep apnea.^33^ Furthermore, the physical limitations of axSpA can affect basic activities such as eating, washing, dressing, and social activities,^34^ as well as the ability to work. Cross‐sectional studies have reported that 10.5%‐32% of patients were forced to give up their jobs due to the disease,^27^, ^28^, ^35^, ^36^, ^37^ with limitations to work ability described by about half of all respondents.^36^, ^37^ Taken together, the evidence shows that axSpA patients face significant challenges, and the main goal of treatment should therefore be to restore and preserve quality of life and normal function for patients as much as possible. It is also important to recognize that the cost of axSpA includes both direct costs related to treatment and indirect costs from loss of work productivity, inability to work, and restrictions in daily function, and all these costs should be considered when optimizing treatment for patients. A cross‐sectional study has shown that axSpA patients who respond to treatment can achieve comparable health‐related quality of life to the general population, and rates of activity impairment (33.3% vs 47.4%, *P* < .001) were also significantly lower than non‐responders, indicating the importance of effective treatment.^24^ When quality of life and normal function are achieved through effective management, patients will require less burden of care, and the overall socioeconomic costs are expected to be lower as well.

**Overarching Principle 3: Optimal management of axSpA requires a range of treatment strategies, including non‐pharmacological treatment, pharmacological treatment, surgery, and lifestyle modification. (LoA: 100%)**

The management of axSpA should not be limited to a single treatment strategy, but likely requires a range of strategies, the combination, sequence, time of initiation, and duration of which may be important for patient outcomes. Non‐pharmacological, pharmacological, and surgical treatments are further discussed in their respective recommendations and accompanying statements, but it is also important for clinicians to recognize that lifestyle modification, such as smoking cessation and regular exercise, can play a key role in management.

Although not part of the four management strategies described above, axSpA patients in Taiwan may seek out complementary and alternative medicine (CAM) such as herbal remedies, acupuncture, moxibustion, and therapeutic massage (eg *tuina*), either voluntarily or on the advice of family and friends. Therefore, it is important rheumatologists be aware that patients are likely to use CAM, and be ready to provide evidence‐based advice insomuch as it is available. No randomized controlled trials (RCTs) with herbal remedies have been conducted in axSpA patients to date, and it should be noted that the use of herbal remedies can vary widely in terms of source ingredients, dose, regimen, and formulation. The quality of such products is not well‐regulated, and rheumatologists should therefore encourage patients to seek out licensed and accredited establishments and practitioners (eg government‐licensed Chinese medicine practitioners in Taiwan) whenever possible. Regarding acupuncture and/or moxibustion, a small randomized trial comparing heat‐sensitive moxibustion (cupping) with acupoint injection vs oral medication controls in 116 AS patients found that cupping and acupuncture relieved major symptoms and improved joint function markedly in 38.9% of test subjects vs 11.9% of control subjects (*P* < .05).^38^ Regarding physiotherapy and therapeutic massage, a 2008 Cochrane review found low to moderate quality evidence to support combined inpatient spa‐exercise therapy followed by group physiotherapy over group physiotherapy alone, and for supervised group physiotherapy over home exercises.^39^ However, any type of spinal manipulation should be avoided in axSpA patients due to the risk of osteoporosis and spinal fracture, and rheumatologists should expressly warn patients of the risk involved, especially with the strong massage and joint manipulation techniques common in *tuina*.

**Overarching Principle 4: Treatment of axSpA should involve shared decision‐making between the patient and health professionals in order to achieve optimal care. (LoA: 100%)**

The goal of axSpA treatment is to enable the patient to secure quality of life and normalized function, but the patient's definition of normal life and function may be significantly different from that of the rheumatologist or other healthcare professionals. Therefore, it is important to work closely with the patient to understand his or her treatment goals, identify and explain potential barriers to achieving those goals, and help the patient make an informed decision regarding treatment options. This process needs to continue throughout the management of axSpA. To achieve shared decision‐making, patients need to be provided with adequate education about their disease, and the risk‐benefit analysis of management decisions also needs to be communicated in a timely and understandable manner. Ideally, shared decision‐making will help to bring patients, caregivers, and healthcare professionals together in working toward optimal care and outcomes.

An important aspect of shared decision‐making for Taiwanese axSpA patients involves family planning and pregnancy management, as many patients are of reproductive age and may face family pressure to conceive.^40^ Family planning discussions can fill a key unmet need for both male and female patients.^41^ Inflammation in axSpA has been associated with reduced sperm motility and impaired testicular function,^42^ and small cross‐sectional studies have reported that a majority of married or sexually active male patients felt that the disease had a negative effect on their sexual life, with issues such as low sex drive, premature ejaculation, sexual dissatisfaction, and impotence more frequently occurring.^43^, ^44^, ^45^ In addition, a 2016 Swedish case‐control study of 388 deliveries among AS patients compared to 1082 matched controls from the general population found that even after adjustment for smoking habits, age, education, and comorbidities, female AS patients had a higher risk of caesarean section, preterm birth, and small‐for‐gestational‐age.^46^ Prior to conception, adjustments to therapy may be needed to avoid miscarriage or congenital abnormalities; however, pregnant axSpA patients can still experience active disease, and therefore treatment may still need to be maintained during pregnancy.^47^ Recommendations for patients with rheumatic diseases on the use of nonsteroidal anti‐inflammatory drugs (NSAIDs),^48^, ^49^, ^50^ conventional synthetic disease‐modifying antirheumatic drugs (csDMARDs),^49^, ^50^ and tumor necrosis factor inhibitors (TNFi)^49^, ^50^, ^51^, ^52^, ^53^, ^54^ during pregnancy and lactation are currently available, but there is insufficient data to make any recommendations regarding the use of IL‐17i at present.^55^

**Overarching Principle 5: The management of axSpA in Taiwan is strongly influenced by the National Health Insurance reimbursement system and local health circumstances. (LoA: 100%)**

In Taiwan, >99% of the population is covered under the National Health Insurance (NHI) program,^56^, ^57^ and therefore the NHI reimbursement criteria has a critical influence on axSpA management. In addition, local health circumstances such as availability of medications, approved indications, prevalence of tuberculosis and hepatitis B, and patient preferences can also affect treatment. The Taiwan NHI program currently recognizes AS, but not axSpA, as a disease indication.^58^ A variety of traditional NSAIDs and cyclooxygenase‐2 (COX‐2) inhibitors are covered by the NHI for the long‐term treatment of inflammation and associated pain in AS patients.^59^ In addition, csDMARDs are reimbursed for the treatment of peripheral symptoms in AS patients.^58^ The NHI currently covers the use of biologics (bDMARDs, including TNFi and IL‐17i) for the treatment of AS, but the reimbursement criteria is quite strict: biologics must be prescribed by clinicians licensed in the fields of rheumatology or immunology, and reimbursement must be applied for and approved prior to treatment initiation. In addition, the patient must be aged 18 or above, be positive for human leukocyte antigen‐B27, have radiographic evidence of sacroiliitis, and demonstrate at least two out of the following three conditions: limitations in lumbar flexion, limitations in chest expansion, or >3 months of lower back pain and morning stiffness that is not relieved by rest but improves with exercise.^58^ Moreover, biologics may be initiated only when patients have persistently high disease activity (BASDAI score ≥6 and erythrocyte sedimentation rate [ESR] >28 mm/1 h and C‐reactive protein [CRP] >1 mg/dL in two consecutive tests, with at least a 4‐week interval in between tests) and fails to respond to extensive treatment with at least two different NSAIDs (must have received continuous treatment at the same clinic or institution for 3 months or more, and must have used each NSAID for at least 4 weeks or more, unless discontinuation due to toxicity or tolerance occurs; those with peripheral symptoms must have undergone extensive treatment with at least two NSAIDs and sulfasalazine).^58^ Furthermore, all patients must present a certificate indicating they have received exercise‐related patient education (or an affidavit stating that they exercise regularly at home), and must sign a treatment consent form indicating they understand the indications, contraindications, and side effects of therapy.^58^ AS patients who receive reimbursed biologics need to undergo BASDAI evaluation after 12 weeks of treatment, and must demonstrate >50% improvement or a decrease of at least two points to continue therapy; efficacy evaluations should subsequently be undertaken every 12 weeks for those who continue treatment.^58^

The endemic presence of tuberculosis^12^ and hepatitis B^13^ in Taiwan is a critical issue. The incidence and prevalence of tuberculosis in Taiwan are both considerably higher than that seen in the USA or Europe.^12^ This can pose a problem, as a recent meta‐analysis of 71 RCTs involving 22 760 patients with AS, rheumatoid arthritis, or psoriatic arthritis found that the use of TNFi was associated with a 20% increase in the occurrence of any infection, a 40% increase in serious infections, and a 250% increase in tuberculosis occurrence (including both reactivation and new infections).^60^ A new generation of biologics targeting the IL‐17 pathway, such as the IL‐17i secukinumab, may represent a possible solution. In a pooled analysis of safety data from three controlled trials of secukinumab in psoriatic arthritis and two trials in AS, involving 1045 psoriatic arthritis patients and 620 AS patients, no cases of tuberculosis activation were recorded.^61^ A subanalysis of 51 Taiwanese patients who participated in the ERASURE phase III study of secukinumab for plaque psoriasis also reported no cases of tuberculosis reactivation or infection, but observed that upper respiratory tract infection was the most common adverse event.^62^ However, it should be noted that real‐world safety data for secukinumab remains limited. For patients scheduled to receive TNFi, the 2012 TRA consensus recommendations for the screening and management of tuberculosis patients note that patients should be screened for active and latent tuberculosis infections prior to treatment, and the possibility of false‐positive screening results should be considered, as almost all Taiwanese have received the bacillus Calmette‐Guerin vaccine, and rates of non‐tuberculosis mycobacterial infection are rising in Taiwan as well.^63^

The burden of hepatitis B in Taiwan is also great,^13^ and it has been reported that the frequency of hepatitis B virus (HBV) carriers in patients with rheumatic disease (including AS, rheumatoid arthritis, and psoriatic arthritis) was much higher in Taiwan compared to European countries, while the use of TNFi was associated with a 39% HBV reactivation rate in HBV carriers.^64^ Hepatitis B reactivation can occur under immunosuppressive conditions,^65^ and the 2012 TRA consensus recommendations regarding the screening and management of hepatitis B infection in rheumatic patients scheduled for biologic therapy recommend screening for HBV using a chemiluminescent immunoassay/chemiluminescent enzyme immunoassay (CLIA/CLEIA) for HBV serology and a real‐time polymerase chain reaction (RT‐PCR) method for HBV‐DNA in all patients slated to receive treatment with biologics or csDMARDs.^66^ If HBV‐DNA levels are detectable, it is recommended to start antiviral therapy first until HBV‐DNA levels are undetectable, and it is also recommended that inactive HBV carriers and those with resolved HBV infections should receive prophylactic antiviral treatment; however, due to the high cost of antivirals such as entecavir, prophylaxis is generally given only after the detection of serum HBV‐DNA or HBV surface antigen (HBsAg) seroconversion.^66^ Once csDMARD/biologic treatment is begun, serum HBV‐DNA and liver transaminase levels should be monitored every month for the first 3 months, and then once every 3 months, so that reactivation can be managed in a timely fashion.^66^, ^67^

## 4 RECOMMENDATIONS

A total of 11 consensus recommendations were formulated, and their LoE, GoR, and LoA have been noted (Table 1). These recommendations are intended to provide guidance for axSpA treatment and treatment‐related issues in clinical practice, and have therefore been designed to be as clinically relevant as possible.

### 4.1 Recommendation 1

**Treatment for axSpA patients should be individualized according to the signs and symptoms of disease, patient characteristics, and treatment goals.**

**(LoE: IV; GoR: D; LoA: 100%)**

This recommendation stems from the overarching principles, and considering that axSpA is a disease with diverse manifestations (axial symptoms, peripheral symptoms, and EAM) that require multiple treatment strategies, rheumatologists should expect that a high level of individualization will be needed in the management of axSpA patients, and plan accordingly for this.

### 4.2 Recommendation 2

**The diagnosis and monitoring of axSpA disease activity should be based on clinical symptoms and signs, laboratory tests, and imaging, while the frequency of monitoring should be decided on an individual basis.**

**(LoE: IV; GoR: D; LoA: 100%)**

In order to diagnose axSpA and track subsequent clinical improvement or worsening, methods of disease evaluation are necessary. The diagnosis of axSpA is based upon the 2009 ASAS classification criteria,^15^ and factors such as new bone formation (syndesmophytes) can be used to evaluate prognosis. In addition, the 2009 ASAS classification criteria allows for diagnosis based on positive findings (eg bone marrow edema) from either X‐ray or magnetic resonance imaging (MRI).^15^ X‐ray imaging can reveal syndesmophytes, but since radiologic progression is slow,^68^ it is suggested that the interval between spinal X‐rays should be no less than 2 years. The modified Stoke Ankylosing Spondylitis Spine Score (mSASSS) can be used to evaluate radiologic changes, but is mostly used for clinical research. MRI can detect early inflammation before structural damage is radiographically visible, and a high degree of spinal inflammation observed on MRI has been correlated with a successful response to TNFi.^69^ Moreover, signs of disease progression (inflammation, bone erosion, fatty change) can be visualized on MRI, and these can be used to support decisions on whether or not an X‐ray to assess the presence of syndesmophytes will be needed.^69^ The main limitations of MRI are the high cost and the risk of non‐reimbursement by the NHI, which will affect examination frequency. Generally, the frequency of monitoring should be decided on an individual basis,^1^ in line with Recommendation 1 of these guidelines. However, patients who have active disease, are receiving biologics, or have undergone recent changes in treatment may need to be more frequently monitored. Ultrasound may sometimes be used in axSpA patients to evaluate peripheral arthritis, enthesitis, and EAM, and can be used for monitoring in such cases; however, ultrasound is not effective for spinal imaging. In addition, ESR and serum CRP are two common laboratory measures of disease activity, and the BASDAI or Ankylosing Spondylitis Disease Activity Score (ASDAS) are frequently used to determine disease severity. A recent cross‐sectional study of 81 axSpA patients showed a good correlation between the ASDAS and MRI inflammatory scores in nr‐axSpA but not in AS^70^; however, another cross‐sectional study of 40 axSpA patients failed to establish correlation between the Spondyloarthritis Research Consortium of Canada (SPARCC) MRI disease activity score and ASDAS or the BASDAI.^71^ Disease function is commonly scored using the BASFI. The use of these scores and laboratory tests should also be determined on an individual basis, and may be used to track progress in patients receiving TNFi or other biologics.

### 4.3 Recommendation 3

**axSpA patients should be treated to the clinical target (T2T) of reaching either clinical remission or at least minimal disease activity (MDA). The MDA for axSpA has not been defined yet, but achieving ASDAS < 2.1 and preferably < 1.3 is recommended.**

**(LoE: IV; GoR: D; LoA: 78.6%)**

In rheumatoid arthritis, T2T is clearly defined as either remission or low disease activity, but this is not the case for axSpA, which also lacks a definition for MDA.^72^ The 2017 update of recommendations by an international task force have stated that treatment targets for axSpA should include clinical remission or inactive musculoskeletal disease and EAM; in addition, imaging results may also be considered in clinical management alongside clinical and laboratory measures.^2^ BASDAI < 4 with normal acute phase reactants is widely accepted as being indicative of low disease activity,^73^, ^74^ while ASDAS < 2.1 is also a commonly used indicator of MDA.^74^ ASDAS < 1.3 (indicative of inactive disease) is also gaining recognition as a treatment target.^2^ Based on the 2016 ASAS‐EULAR recommendations,^1^ clinical remission in axSpA is considered to be when all clinical symptoms and signs are absent, and levels of inflammatory markers (eg ESR, CRP) are normal. In these guidelines, after taking into account the fact that ASDAS scores were found to be more responsive to clinical measures such as CRP levels, MRI sacroiliac joint inflammation scores, and MRI total inflammation scores,^75^ the MDA for axSpA is recommended to be ASDAS < 2.1 and preferably ASDAS < 1.3. It should be noted that this recommendation does not override the need to help the patient achieve his or her desired treatment objectives; indeed, it is expected that the achievement of T2T will facilitate this outcome. However, the guideline committee also notes that with no local Taiwan data available, and with the NHI reimbursement criteria strongly influencing clinical management, the achievement of even ASDAS < 2.1 may be challenging. Moreover, treatment targets for axSpA may need to be flexibly adjusted according to disease duration, as ASDAS appears to correlate with radiographic progression mostly during the early years of disease.^70^, ^76^, ^77^

### 4.4 Recommendation 4

**Patients with axSpA should be encouraged to stop smoking and start an individualized regular exercise program as soon as possible. The program should emphasize flexibility training, especially spinal mobility exercises, but aerobic exercise, resistance training, breathing exercises, and physiotherapy are also recommended.**

**(LoE: IIa; GoR: B; LoA: 92.9%)**

A recent study from Taiwan observed a strong association between smoking and poor disease prognosis in AS patients,^78^ and it has also been reported that axSpA patients who smoke have a reduced response to TNFi treatment^79^ and greater disease activity.^80^ Therefore, all axSpA patients should be actively encouraged to stop smoking upon diagnosis, and smoking cessation programs may facilitate this. Regular exercise is recommended in axSpA patients, and is associated with benefits such as improved joint mobility, reduced disease activity, and decreased cardiovascular (CV) risk.^81^, ^82^, ^83^ It is important that exercise and physiotherapy programs be individualized so as to improve adherence and minimize the risk of injury.^84^, ^85^ AS patients often have fragile and osteoporotic spines that can develop spinal fractures or dislocations from only mild trauma, and therefore contact sports, cervical traction, and spinal manipulation should be avoided. Exercise programs should include three components: flexibility training (especially spinal mobility exercises; may be performed every day), resistance training (may be performed 2‐3 days per week), and aerobic exercise (preferably swimming, other aquatic exercise, riding a stationary bicycle, or walking on a flat surface; may be performed 3‐5 days per week or for a total of 150 minutes per week at moderate intensity).^86^ A warm‐up of 5‐10 minutes is recommended before exercise, as is a cool‐down of about 5‐10 minutes after exercise, and intensity levels should be increased gradually, in order to minimize the risk of injury.^86^ It is also important to avoid any intense strenuous exercise when there is a flare‐up or acute inflammation.^86^

Postural education and exercise are important to help patients avoid positions that lead to prolonged stooping.^86^ In addition, daily deep breathing exercises with emphasis on full rib cage expansion are recommended to improve breathing patterns, as inflammation of the costochondral joints, costovertebral joints, or entheses can cause chest pain and prevent deep breathing.^86^ Physical modalities such as hot or cold packs, hot baths, hydrotherapy, electrotherapy, diathermy, spa therapy, and mobility exercise may also be considered, as they can help to alleviate pain and may be beneficial for axSpA patients who have gastrointestinal issues and are thus unable to use NSAIDs. These recommendations are largely in line with the 2017 NICE guidance,^3^ which recommend that axSpA patients should be referred to a physiatrist or a physiotherapist to start a structured exercise program, or referred to other specialists (eg occupational therapist, orthotist, podiatrist, etc) when difficulties with daily activities emerge. A 2016 evidence‐based consensus statement regarding exercise in AS patients further recommends that exercises with an emphasis on improving or maintaining spinal mobility, such as specific proprioceptive neuromuscular facilitation techniques, are critical for advanced AS (known as “bamboo spine”) patients, and stretching, strengthening, cardiopulmonary, and functional fitness exercises are also important components of a balanced exercise program for such patients.^85^ There is also preliminary evidence that suggests modified Pilates and Tai Chi, incentive spirometry, and global postural re‐education may be effective; a controlled trial conducted in 40 AS patients found that 60 minutes of Tai Chi twice weekly for 8 weeks followed by 8 weeks of home‐based Tai Chi with a Tai Chi video improved disease activity and flexibility over controls who had no structured exercise program.^87^

### 4.5 Recommendation 5

**EAM are an important part of axSpA and should be actively evaluated and managed to improve patient outcomes.**

**(LoE: IV; GoR: D; LoA: 92.9%)**

A significant proportion of axSpA patients will experience EAM and other comorbidities, and these can have a serious impact on physical function and quality of life. Meta‐analysis results have shown that EAM incidence is comparable in nr‐axSpA and AS patients,^18^ indicating that EAM is a valid concern even in patients with relatively mild disease. Uveitis is the most common EAM by far, with a reported pooled prevalence rate of 21.4% in Asian AS patients.^19^ Acute anterior uveitis is the most frequent type associated with AS, and should be managed as an emergency to avoid complications; a Taiwanese study of 146 AS patients has also reported that acute anterior uveitis is associated with higher disease activity and poor functional ability.^88^ Generally, axSpA patients with anterior uveitis should be referred to an ophthalmologist as soon as possible, as inflammation can lead to papillary and lens dysfunction with blurring of vision, and glaucoma and severe impairment of vision can occur in some cases if adequate treatment is delayed.^89^, ^90^ However, the prognosis is generally good following treatment with topical mydriatics, cycloplegics, and corticosteroids, and dilating drops (eg scopolamine) may be used to relieve pain.^89^, ^90^ Most acute anterior uveitis cases resolve spontaneously within 3 months.^90^ It has been reported that sulfasalazine can reduce the incidence of uveitis flares^91^, ^92^ and the intensity of new flares,^92^ and TNFi have been confirmed to reduce acute uveitis flares in AS patients as well.^93^, ^94^, ^95^, ^96^ However, although meta‐analysis^93^ and retrospective study^94^ results indicated that etanercept, infliximab, and adalimumab were all effective in reducing acute flares, it was noted that infliximab^94^ and adalimumab^94^, ^95^ may be more effective, while etanercept may be no better than placebo.^96^ Psoriasis is also a common EAM, with pooled prevalence reported to be 3.1% in Asian AS patients.^19^ TNFi (etanercept, infliximab, adalimumab) and IL‐17i (secukinumab approved; ixekizumab and brodalumab currently under investigation) are indicated for AS and psoriatic arthritis and/or psoriasis in many countries^89^, ^90^; however, 1.5%‐5% of AS patients may experience onset or worsening of psoriasis following etanercept treatment,^97^ and a prior history of psoriasis was listed as one of the key risk factors for this paradoxical phenomenon. It has been reported that 50%‐60% of AS patients demonstrate histological gut inflammation, and remission of joint inflammation consistently associates with the disappearance of gut inflammation.^98^ Unchecked gut inflammation can develop into IBD (including Crohn's disease and ulcerative colitis), for which the pooled prevalence in Asian AS patients has been reported to be 2.9%.^19^ For IBD patients, gut inflammation conditions can be exacerbated by NSAIDs, which should therefore be used intermittently in low to moderate doses under close monitoring in consultation with a gastroenterologist.^89^ csDMARDs, including methotrexate, azathioprine, or sulfasalazine, have been reported to be effective against IBD,^99^ and in Taiwan, several TNFi are indicated for the treatment of Crohn's disease (infliximab, adalimumab) or ulcerative colitis (infliximab, adalimumab, golimumab)^58^, ^90^; however, etanercept^100^ and the IL‐17i secukinumab^101^ were not found to be effective in the treatment of Crohn's disease. A study of 11 701 Taiwanese AS patients has observed a greater risk of comorbidities compared to the general population,^21^ and EAM in the lung, kidney, and heart can also develop.^102^ EAM in the lung typically include interstitial lung disease, apical fibrosis, and bronchiectasis, but the underlying pathophysiology remains unclear. It has been reported that up to 52% of AS patients demonstrate some type of lung involvement on high‐resolution computed tomography (CT) scan.^103^ Considering that many patients may have pathological lung changes that will not be discovered unless CT or biopsy tests are conducted, it is suggested that clinicians can be more proactive and conduct these tests to assess the presence and severity of lung EAM in axSpA patients when necessary. Although the prevalence of kidney EAM in AS patients is low, many patients (10%‐35%) will have renal comorbidities such as amyloidosis and IgA‐associated nephropathy,^21^, ^90^, ^104^ which can significantly increase mortality risk. Evidence pertaining to treatment is limited to a report describing possible benefit for amyloidosis with long‐term (>1 year) etanercept treatment.^105^ It has been reported that 10%‐30% of AS patients have heart pathologies (encompassing EAM and comorbidities), and the inflammatory environment of axSpA is known to increase CV risk in patients; this is a key cause of mortality.^106^, ^107^ TNFi may help to reduce inflammation and CV risk, but no direct evidence is available to date. It should be noted that NSAIDs can exacerbate CV risk both during treatment and within the immediate weeks after treatment cessation,^108^ and decisions related to continuous or on‐demand NSAIDs should be taken with this issue in mind. Further information regarding NSAIDs is available in the statement accompanying Recommendation 6.

### 4.6 Recommendation 6

**NSAIDs are the first‐line treatment to ensure symptom control for symptomatic axSpA, and it is recommended to use an optimal dose to minimize complications. Ongoing monitoring of renal function, as well as gastrointestinal and cardiovascular side effects, should be determined on an individual basis. Analgesics may be considered to treat residual pain.**

**(LoE: Ia; GoR: A; LoA: 92.9%)**

Nonsteroidal anti‐inflammatory drugs remain the first‐line treatment for symptomatic axSpA patients, and it is recommended to use an optimal dose to ensure symptom control and minimize complications. Renal function may need to be monitored in patients receiving NSAIDs, to be determined on an individual basis. The potential to increase gastrointestinal toxicity and CV risk are the two most commonly cited issues with NSAID use. Several RCTs have shown that COX‐2 inhibitors have reduced gastrointestinal toxicity as compared to traditional NSAIDs.^109^ Moreover, in patients taking celecoxib 400 mg/d for 6 months, gastric ulcer risk was reduced by more than 70% with the addition of a proton‐pump inhibitor.^110^ Therefore, when selecting NSAIDs in patients with gastrointestinal risk factors, COX‐2 inhibitors are preferred, and the use of gastro‐protective treatment should be considered.

Regarding CV risk, a long‐term placebo‐controlled trial of celecoxib 400 and 800 mg/d (a dose not used in rheumatology practice) for the prevention of colonic adenomas demonstrated increased risk of CV events with treatment.^111^ By contrast, celecoxib at 400 mg/d did not increase CV risk in two other long‐term placebo‐controlled trials, for the prevention of adenomatous polyps^112^ and Alzheimer's disease.^113^ In addition to these reports, two large meta‐analyses comparing NSAIDs with placebo treatment revealed that COX‐2 inhibitors, diclofenac, and ibuprofen all increased major vascular events to varying degrees, while only naproxen did not increase either major vascular events or mortality.^114^, ^115^ It should be noted that, compared to patients with autoimmune diseases, the patients recruited in the above trials did not have diseases with persistent inflammation. Given that chronic inflammation is recognized as a CV risk factor, the anti‐inflammatory effect of NSAIDs may reduce the CV risk of autoimmune diseases, and such an effect might counterbalance the increased CV risk from NSAID treatment in arthritis patients. Several trials have been performed in patients with rheumatoid arthritis or osteoarthritis, but the immunomodulatory effects of csDMARDs cannot be excluded from the study results. In the CLASS (Celecoxib Long‐Term Arthritis Safety Study) trial, celecoxib 800 mg/d did not carry greater CV risk than diclofenac 150 mg/d or ibuprofen 2400 mg/d.^116^ Recently, the Prospective Randomized Evaluation of Celecoxib Integrated Safety vs Ibuprofen or Naproxen (PRECISION) trial, which was conducted to assess the cardiovascular risk of celecoxib 200 mg/d as compared with traditional NSAIDs (ibuprofen 1800 mg/d and naproxen 750 mg/d) in patients with rheumatoid arthritis or osteoarthritis, found celecoxib to be non‐inferior to ibuprofen or naproxen with regard to CV safety, and naproxen did not demonstrate superior CV outcomes over celecoxib or ibuprofen.^117^ A sub‐study of the PRECISION trial further found that a lower percentage of celecoxib users developed hypertension compared to ibuprofen or naproxen users.^118^ Moreover, in a recent case‐control study of 421 AS patients with CV disease (CVD) and 842 sex‐ and age‐matched controls collated from the Taiwan NHI claims database for the period spanning 1997‐2008, it was found that although AS patients are at increased risk of CVD, frequent COX‐2 inhibitor users had a 10‐fold lower CVD risk at 24 months compared to non‐users.^119^ Another recent 10‐year population‐based retrospective cohort study encompassing a total of 1208 AS patients and 19 328 non‐AS patients sampled from the Taiwan NHI claims database similarly found that high cumulative defined daily doses of celecoxib had significant protective effects against CVD in AS and control patients.^120^ Regarding non‐frequent NSAIDs users, although short‐term exposure to NSAIDs did carry higher CVD risk, this risk disappeared after 12 months, suggesting that long‐term NSAID use may alleviate CV risk in AS patients. This result was mirrored by two other studies,^121^, ^122^ which showed that lack of exposure to NSAIDs was a risk factor for vascular mortality in AS patients. Considering that individual CVD risk is influenced by a number of factors, including age and pre‐existing CV risk, the use of NSAIDs should be tailored on a case‐by‐case basis.

Regarding long‐term NSAID use in AS patients, the key question is whether treatment carries increased risk, and the risks and benefits of continuous or on‐demand NSAIDs should be critically evaluated. A 2‐year randomized controlled trial comparing continuous and on‐demand NSAID treatment found that continuous celecoxib use reduced radiographic progression in AS patients.^123^ Similar results were observed in a retrospective analysis of the German Spondyloarthritis Inception Cohort, which compared high and low NSAID intake over 2 years.^124^ Patients with elevated CRP or high disease activity appear to benefit more from continuous NSAID use^124^, ^125^; however, this was challenged in a recently published randomized controlled trial, which found that continuous use of diclofenac over 2 years did not reduce radiographic progression in AS patients compared to on‐demand diclofenac, even when subgroup analysis of patients identified as being more susceptible to radiographic progression (ie patients with elevated CRP, baseline syndesmophytes, or smokers) was conducted.^126^ Further research will be needed to confirm that continuous NSAID use can delay radiographic progression in AS, and the type of NSAIDs used may affect outcomes as well. Therefore, it is recommended that continuous use of NSAIDs should be guided by patient symptoms and objective measures of inflammation, rather than by the treatment goal of preventing structural progression.^1^ The risks and benefits of NSAIDs should be continuously evaluated throughout treatment, and if symptoms recur after stopping or reducing the dose of an NSAID, continuous use is advised.^1^ It should be noted that NSAIDs are not primarily prescribed for the alleviation of pain, but for the control of inflammation; any residual pain can be managed through conventional analgesics. Although formal evidence that analgesics are efficacious in axSpA is lacking, the 2016 ASAS‐EULAR guidelines recommend that analgesics, such as paracetamol and opioid‐like drugs, might be considered for residual pain after previously recommended treatments have failed, are contraindicated, or are poorly tolerated.^1^

### 4.7 Recommendation 7

**Local injections of glucocorticoids to sites of inflammation and short‐term systemic glucocorticoids may be beneficial, but long‐term treatment with systemic glucocorticoids should be avoided.**

**(LoE: IIa; GoR: B; LoA: 85.7%)**

Long‐term treatment with systemic glucocorticoids is not recommended in the treatment of axSpA patients; however, a recent study has found that the short‐term use of high‐dose (50 mg/d) oral prednisolone in active AS patients was more effective in achieving 50% of BASDAI improvement after 2 weeks of treatment, compared to a 20 mg/d dose or placebo.^127^ This suggests that a daily dose of 50 mg oral prednisolone could be considered as a short‐term bridging solution or flare treatment. The local use of image‐guided glucocorticoid injections at sites of musculoskeletal inflammation is still recommended^128^, ^129^; however, uncontrolled overuse of local injections should be avoided.

### 4.8 Recommendation 8

**Although csDMARD monotherapy is not recommended for axSpA, it can be effective against peripheral arthritis and EAM; co‐administration of csDMARDs with biologics may be beneficial in axSpA, but further evidence is needed to confirm this.**

**(LoE: IIa; GoR: B; LoA: 85.7%)**

Evidence for the use of csDMARD monotherapy in the treatment of axSpA remains inconclusive, and controlled studies involving sulfasalazine,^130^, ^131^, ^132^, ^133^ mesalazine,^134^, ^135^, ^136^ methotrexate,^131^, ^137^ or leflunomide^138^, ^139^ in AS or axSpA patients failed to consistently observe significant improvement in BASDAI, BASFI, or other measures of disease activity. Therefore, csDMARD monotherapy is not recommended for the treatment of axSpA per se.^1^, ^130^, ^131^, ^132^, ^137^, ^140^ However, sulfasalazine,^140^, ^141^ and leflunomide^138^ may be beneficial against peripheral arthritis, and csDMARDs have shown efficacy in the treatment of EAM; for example, it has been reported that sulfasalazine can reduce the incidence of uveitis flares^91^, ^92^ and the intensity of new flares,^92^ and methotrexate, azathioprine, and sulfasalazine have been reported to be effective against IBD.^99^ High cumulative defined daily doses of sulfasalazine have also been shown to significantly reduce CV risk in a large Taiwanese retrospective cohort study.^120^ Interestingly, recent studies have shown that methotrexate may play a role in reducing immunogenicity and anti‐drug antibody formation when administered concomitantly with TNFi (adalimumab,^142^, ^143^ infliximab^143^). A recent cohort study of 1365 AS patients and 1155 undifferentiated spondyloarthritis patients further found that patients who used csDMARDs concomitantly with TNFi (adalimumab, etanercept, or infliximab) had better 5‐year retention for their first TNFi.^144^ These results point to possible benefit with co‐administration of csDMARDs during TNFi treatment, but further evidence will be needed to confirm this.^145^

### 4.9 Recommendation 9

**In the event of treatment failure with conventional therapy, after evaluating other causes, biologic therapy should be considered for axSpA.**

**(LoE: Ia; GoR: A; LoA: 92.9%)**

In the event of treatment failure with conventional therapy, other causes should be evaluated and addressed first, including but not limited to osteoporosis, spinal fractures, malignancy, fibromyalgia, tuberculosis, or tuberculosis spine. It should be noted that the risk of osteoporosis and spinal fractures in axSpA patients is significant even in the early stages of disease^20^; however, osteoporosis is generally asymptomatic until fractures develop, and for axSpA patients with unknown persistent back pain other than that known to be associated with inflammation, the possibility of spinal fracture should be considered and assessed. The prevalence of spinal fractures in AS has been reported to be 10%‐40%, with a significantly increased risk of fracture as compared to the general population^20^; moreover, following spinal fracture, patients with AS are 11 times more likely to sustain a spinal cord injury than the general population.^146^ Despite this risk, epidemiology reports suggest that more than half of axSpA patients have never undergone any kind of bone densitometry test,^147^ while in those tested, more than half are regularly found to have low bone mineral density.^148^ Considering that bicycles and motorcycles are often used for transportation in Taiwan, the risk of osteoporosis and spinal fracture needs to be addressed as early as possible in axSpA patients, to prevent complications or even permanent disability arising as the result of an accident or fall while riding a bicycle or motorcycle.

Once other causes have been evaluated and excluded, biologic therapy may be considered. There are currently two main classes of biologics available for the treatment of axSpA (AS): TNFi (including etanercept, infliximab, adalimumab, certolizumab, and golimumab) and IL‐17i (secukinumab approved; ixekizumab and brodalumab currently under investigation). In Taiwan, the NHI currently covers adalimumab, etanercept, golimumab, and secukinumab for the treatment of AS.^58^ The European Medicines Agency has approved the use of TNFi for the treatment of nr‐axSpA patients, but the US Food and Drug Administration has not approved any therapeutic indications for axSpA or nr‐axSpA as yet, citing difficulties in defining the disease and monitoring treatment efficacy. Although the availability of biologics may vary between countries, in the event that both classes are available, the choice of biologic should be made according to disease severity, EAM, comorbidities, and other characteristics of individual patients.^1^ In current practice, biologics are usually initiated with TNFi treatment, but there may be instances for which IL‐17i might be a better starting biologic: for example, tuberculosis or HBV reactivation following TNFi treatment is a major concern, and the IL‐17i secukinumab appears to have lower risk of tuberculosis reactivation,^61^, ^62^ although long‐term real‐world safety data will be needed to confirm this.

### 4.10 Recommendation 10

**Intra‐ or inter‐class switching between biologics or small molecule therapies may be considered for patients with inadequate response or who become intolerant to therapy.**

**(LoE: Ia; GoR: A; LoA: 92.9%)**

Previously, only one class (TNFi) of biologics was available for the treatment of axSpA, but with the advent of IL‐17i and other agents targeting the IL‐23/IL‐17 axis,^149^ intra‐ or inter‐class switching between biologic agents is now possible for patients who have inadequate response or become intolerant to therapy. A recently published systematic review of 134 studies found that the main reasons that induced switching from initial TNFi therapy included lack of efficacy (14%‐68%; primary failure), adverse events/poor tolerability (13%‐57%; primary failure), and loss of efficacy (13%‐61%; secondary failure).^150^ For cases of primary failure characterized by lack of response, it may be worthwhile to re‐consider the diagnosis, as the ASAS‐EULAR guideline task force has stated that true primary failure is rare in axSpA patients with active disease^1^; however, patients with true primary failure or intolerance/toxicity to TNFi may benefit from switching to IL‐17i.^1^ For patients with secondary failure characterized by loss of response, intra‐class switching can extend efficacy, but it should be noted that drug survival rates are generally lower for the second (47%‐72%) and third (49%) TNFi at 2 years.^150^, ^151^ In patients who lose response to TNFi, switching to an IL‐17i can be beneficial, but overall efficacy may be less than in TNF‐naïve patients.^152^ Switching for primary failure may be conducted after the patient has initiated one class of therapy and found to be unresponsive or intolerant within 3‐6 months, while switching for secondary failure may be conducted if regular monitoring shows that response to treatment has decreased, or if anti‐drug antibodies emerge.^1^, ^150^ As small molecule therapies emerge in the near future, it may be possible to consider switching to such therapies in the advent of treatment failure with biologics.

A recent study of 42 axSpA patients who underwent dose reduction of TNFi for 1 year found that 76.2% remained in remission or low disease activity at the end of the study, with shorter duration of remission before dose reduction, shorter duration of treatment with biologics, and shorter disease duration found to be risk factors for relapse.^153^ Tapering or dose reduction of etanercept has been conducted successfully in AS patients with >6 months of stable disease, with acceptable efficacy in cases that needed to restart a full dose of treatment^154^, ^155^; however, in stable patients who completely discontinued etanercept^156^ or adalimumab,^157^ a greater percentage experienced disease flares as compared to controls. These results indicate that dose reduction may be carried out successfully in long‐term stable patients,^158^ but evidence for the specific timing of dose reduction and the effect of restoring full doses in relapsed patients remains limited for now.

### 4.11 Recommendation 11

**In patients with refractory pain or disability and radiographically visible structural damage of the hip joint, hip arthroplasty should be considered, while corrective osteotomy may be considered for patients with disabling spinal deformity.**

**(LoE: III; GoR: C; LoA: 100%)**

A study of three real‐world datasets involving 2718 AS patients found that 24%‐36% of patients presented with clinically significant hip involvement, and 5% of all pooled patients required hip surgery.^159^ Patients who demonstrate radiographic structural damage and accompanying symptoms should be considered for hip arthroplasty regardless of age, and cementless prostheses are preferred in young patients.^1^ Corrective osteostomy is highly specialized and should be undertaken in consultation with a spinal surgeon for patients with severe and disabling spinal deformities. Two recent retrospective studies respectively involving 13^160^ and 12^161^ AS patients found that kyphosis correction and alleviation of back pain could be achieved by osteotomy through the pathological fracture gap or same‐level transpseudarthrosis osteotomy with surgical repair via interbody fusion by a single posterior approach, indicating that these approaches are feasible and potentially effective.

## 5 DISCUSSION

**Figure 1:**
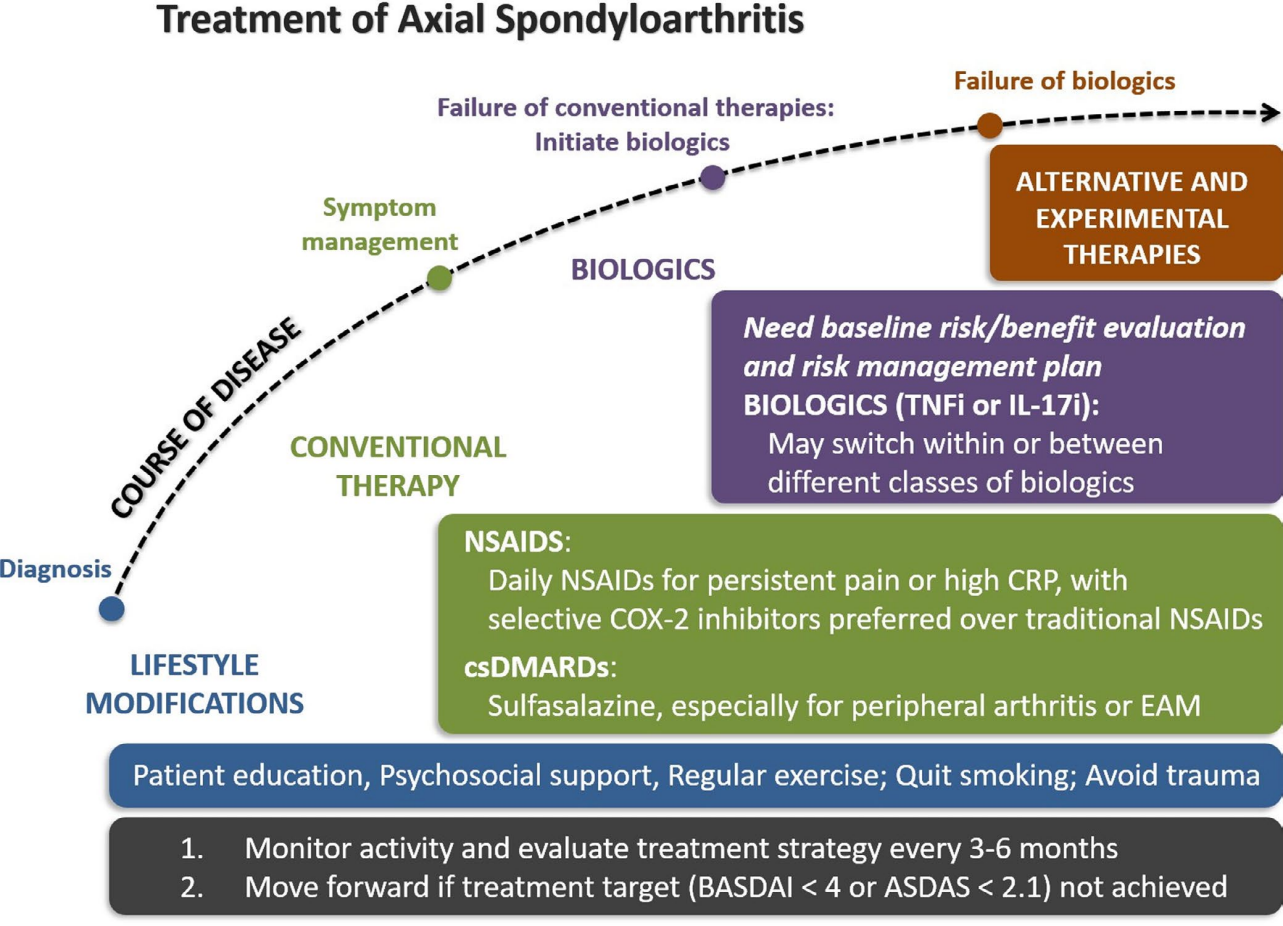
Management algorithm for axSpA

These guidelines have endeavored to include the latest evidence concerning the management of axSpA, and have also incorporated issues of local relevance to clinicians in Taiwan. A clinical algorithm covering key aspects of axSpA management is provided in Figure 1 for easy reference. It should be noted that the Overarching Principles and Recommendations should be read with the accompanying statements to derive the best picture of current evidence, and the listed references may need to be consulted when more information is needed. Although significant challenges still remain with the recognition of axSpA as a category of disease that can be used in drug indications and reimbursement criteria, initiatives aimed at increasing clinician acceptance, promoting related research, and advising healthcare policymakers will be undertaken in the near future. These guidelines will continue to be updated in the event of new evidence or the approval of new treatments for axSpA, and feedback will be actively solicited from rheumatologists, clinicians of other disciplines, other healthcare professionals (including pharmacists, physical therapists, and nurses), professional societies, patient groups, healthcare institutions, regulatory authorities, health insurance providers, and the pharmaceutical industry, in order to ensure that these recommendations remain up‐to‐date and relevant for clinical practice.

## AUTHOR CONTRIBUTIONS

All authors were involved in the discussions and formulation of the recommendations. All authors reviewed and commented extensively on the manuscript. All authors approved the final version of the manuscript.
